# Conflicts and protests of Argentinean nursing during the covid-19 pandemic

**DOI:** 10.1590/S0104-59702023000100060

**Published:** 2023-11-10

**Authors:** Karina Ramacciotti, Adriana María Valobra

**Affiliations:** i Investigadora principal, Departamento de Ciencias Sociales/ Universidad Nacional de Quilmes/Consejo Nacional de Investigaciones Científicas y Técnicas. Ciudad Autónoma de Buenos Aires − Argentina. karinaramacciotti@gmail.com; karinaramacciotti@gmail.comkarinaramacciotti@gmail.com; ii Investigadora principal, Centro Interdisciplinario de Investigaciones en Género/ Universidad Nacional de La Plata/Consejo Nacional de Investigaciones Científicas y Técnicas. La Plata − Argentina indivalobra@gmail.com; indivalobra@gmail.comindivalobra@gmail.com

**Keywords:** Nursery, Pandemic, Labor conflicts, Health policies, Enfermería, Pandemia, Conflictos laborales, Políticas sanitarias

## Abstract

The interest of this article is to study how the covid-19 pandemic, by intensifying work routines, enhanced structural conflicts in the nursing sector of Argentina. For this purpose, we use a quantitative and qualitative methodological strategy that allows us to understand in depth the practices and representations of nurses during the pandemic by means of a self-administered survey and in-depth interviews. This proposal will have two axes. First, we will analyze the conflicts that occurred due to work overload and lack of supplies for protection against covid-19. Secondly, we will review the strategies of collective claims through unions and self-organized movements.

## Resumen

El interés de este artículo es estudiar cómo la pandemia de la covid-19, al intensificar rutinas laborales, potenció conflictos estructurales en el sector de enfermería en Argentina. Para ello, utilizamos una estrategia metodológica cuantitativa y cualitativa que nos permite comprender en profundidad las prácticas y las representaciones de enfermeros y enfermeras durante la pandemia a partir de la realización de una encuesta autoadministrada y entrevistas en profundidad. En primer lugar, analizamos los conflictos acontecidos por la sobrecarga laboral y la falta de insumos para la protección contra la covid-19. En segundo lugar, revisamos las estrategias de reclamos colectivos por medio de sindicatos y de movimientos autoconvocados.

After the appearance of the first case of covid-19 in Argentina (March 3, 2020), the government of Alberto Fernandez limited the movement of people as of March 19 by means of the Preventive and Obligatory Social Isolation (Aislamiento Social Preventivo y Obligatorio, ASPO). During the first months, only “essential” workers were allowed to move, and this category encompassed health personnel, including nurses.

The nursing sector constitutes a key pillar within the healthcare system. According to official sources, at the international level, “nursing is the largest occupational group in the health sector, representing approximately 59 percent of the health professions” (East, Laurence, López Mourelo, 2020). In Argentina, the healthcare sector, moreover, is feminized as an estimated 74% are women. Among these, 85% of the nursing staff are women.^1^

During the covid-19 pandemic, nurses played an active role in tasks of screening, swabs, care, communication with family members and vaccination campaigns. Symbolic signs of recognition expressed through some manifestations of gratitude such as applause at 9 p.m., military bands playing popular songs in hospitals, delivery of awards, gifts and taking selfies that circulated in networks, were not enough to silence the labor conflicts that the sector went through. Thus, in this article, from the crossing of quantitative and qualitative data and the national press survey between March 2020 and July 2021, we will analyze in first place, the conflicts occurred due to work overload and the shortage of supplies for protection against covid-19. And secondly, the demands organized in unions and self-organized movements.

We would like to emphasize that the scenario of claims did not occur in a vacuum but within the framework of a critical situation in socioeconomic terms. The pandemic, in any case, revealed problems and challenges inscribed in both historical and structural processes. Indeed, within a certain consensus regarding the tasks of the nursing sector, once the news of the effects of covid-19 on the health system spread, restrictive measures were implemented and different situations of conflict arose. The demands brought to light historical problems of the profession such as deficient working conditions, labor-intensive processes and times, low salaries and a consequent permanent lack of personnel to cover the growing demand of the sector (Aspiazu, 2017; Pereyra, Micha, 2016; Ramacciotti, Valobra 2017; Ramacciotti, 2020). All this becomes more complex in a context of a health system having different levels of care and, therefore, of coordination of health policies, as well as of the budget available for their implementation which, during periods of implementation of neoliberal policies, intensifies this transfer of responsibilities to the sectors of less regional scope, which are those with the least room of manoeuvre in terms of resources. The previous national government administration, headed by Mauricio Macri of the coalition Cambiemos (2015-2019), had implemented a policy that benefited concentrated financial sectors of the economy, which resulted in a regressive redistribution of income of the middle and lower sectors (Canelo, 2019), and in the reduction of public spending, which implied the withdrawal of the State from areas once considered of capital importance. A key event, which disrupted Argentina’s historical position of State responsibility for health, was the downgrading of the Ministry of Public Health, which became a secretariat under the Ministry of Social Development, with the consequent loss of autonomy and a significant reduction in budget allocations. In December 2019, in this situation, Alberto Fernández took office as president, hand in hand with a Peronist coalition called Frente de Todos. After only three months in office, the health response to covid-19 had to be organized. Health was placed as a central topic in the government agenda. According to the words of the president, in a speech on national TV on April 2, which had the support of the governors: “We can come back from an economic crisis, though death is irreversible: there is no way back from it” (Cibeira, 3 abr. 2020). With this statement, he tried to counteract pressures from powerful economic and political actors and certain media, who maintained, in view of the ASPO decision, the importance of prioritizing the economy before health, and also sought to differentiate himself from the authorities of those countries that minimized the danger of the disease and maintained more lax measures of restriction to circulation.^2^

The quantitative and qualitative methodological strategy is based on a self-administered survey and interviews allowing us to understand in depth the practices and representations of nurses during the pandemic. Both the survey and interview forms were approved by the Ethics Committee of the Posadas National Hospital and were carried out in accordance with current regulations and ethical standards. The corpus is made up of 274 interviews conducted between April and July 2021 with nurses from all over the country, from the public and private sectors, who were in practice during the pandemic. The framework for these conversations is the PISAC COVID-19 0022 Project: “Nursing and professional health care during the covid-19 pandemic and post-pandemic”, in which participated researchers from 16 nodes (national universities and research centers in different regions of the country). The research team is characterized by its interdisciplinary nature, with professionals from the fields of history, sociology, nursing, social work, political sciences, social communication, anthropology, psychology, international trade, occupational therapy, literature, social sciences, geography and health education. This interdisciplinary and federal composition made it possible to enhance the work and make it more dynamic on the basis of broader dialogues than those imposed by the disciplines themselves and to have a set of data in real time on one of the emerging aspects of the covid-19 health crisis in Argentina. Our perspective assumes a point of view that, without blending with the social and political subject that is defined in the research, research is not only for academic purposes but also for reparation. Indeed, as research on the subject has made clear, nursing is a subordinate sector within the health structure, an aspect that cannot be separated from its gender condition. In this sense, without wanting to romanticize the sector, we are not indifferent to its situation and we understand its claim not only in the current situation but also as a structural historical problem, which our research has contributed to make visible. In short, our bet from a feminist theory allows us to recognize our starting point, crossed by subjectivity and sensitivity to this issue, but submitting our position to a constant exercise of reflexivity and strategies of rupture. Therefore, we propose to rethink the idea of objectivity in the construction of knowledge not from the assumption of an aseptic cognitive subject but from the recognition of a positioned subject producing a situated knowledge (De Martinelli, Queirolo, Valobra, 2022).

To determine the number and profiles of interviews and the survey to be conducted in each region, a theoretical sampling by quotas was carried out based on the nursing profiles described in the official report of 2020, made by the National Ministry of Health on the state of the situation of nursing education and professional practice (Ministerio de Salud, 2020). Quotas were defined on the basis of two main variables: the level of training of nurses (assistants, technicians, and graduates)^3^ and the subsector of the establishment where they work (public or private). The recruitment of participants was carried out through a “snowball” strategy based on contacts from the research team and through dissemination on social networks. For the interviews, a semi-structured guide was used which, given the context of the pandemic restrictions, was implemented in a virtual format between April and July 2021, that is, during the second wave of coronavirus contagions in Argentina. The interviews had a duration of 60 to 120 minutes, were recorded and transcribed with the prior consent of the interviewees, whose identity is protected. In addition, a distribution by age and gender was sought to reflect the composition of the labor force employed in this sector.

Regarding labor insertion, people interviewed worked during the pandemic in public administration settings such as provincial, trade unions and municipal hospitals, rapid construction hospital buildings designed to contain emergencies, geriatric hospitals, testing centers, vaccination posts and fever units.

The survey – self-administered, national, anonymous and confidential – was conducted during the months of May and June 2021. That is, during the period in which the highest number of infections occurred – about 30,000 in those two months, a higher figure than that recorded in the first wave in 2020 in the same period – and when the vaccination campaign against covid-19 was not at an advanced stage. A non-probabilistic sample of 1,483 cases was obtained from nurses in all Argentinian provinces. The database contains information on a multiplicity of indicators, which were investigated on the basis of 104 questions distributed in thematic nuclei: sociodemographic data, characteristics of work insertion, processes, times and organization of work, hygiene and safety conditions and work environment, psychosocial and emotional aspects related to work during the pandemic. The data obtained from the survey were systematized and analyzed using SPSS statistical software.

## Work overload and shortage of supplies

Since the confirmation of the first case and the sanitary restrictions imposed, health units had to prioritize the care of patients affected by covid-19, suspend all scheduled consultations and attend only emergencies. This reduction in traffic and in the care of people did not imply greater peace of mind for health personnel. On the contrary, these were days of great uncertainty for health teams, given that this was a new disease, in an unpredictable scenario, characterized by a lack of knowledge about how the pandemic would evolve and doubts about how the system could cope with such a situation. Slowly, with the increase in the number of infections, added to acute peaks of high mortality, the workload increased. For some regions such as the metropolitan area of Buenos Aires,^4^ the overload of tasks and the amount of time required to care for a patient were already topics of discussion in the sector before the pandemic (Pereyra, Micha, 2016; Aspiazu, 2017). Since covid-19, this situation has increased and generated new conflicts (Novick et al., 2020). In this regard, there was a strong response to our survey question “Since the beginning of the pandemic, have activities increased that you did not do before?” with more than 92% of affirmative answers.

The first emerging conflicts in the workplace were related to the lack of personal protective equipment for dealing with the disease. Such is the case of the workers of the Ramos Mejía Hospital in the City of Buenos Aires, who, through a judiciary collective protection order, urgently requested the provision of the elements and supplies recommended by the World Health Organization (WHO), the National Ministry of Health and the Superintendence of Occupational Risks. In detail, the request included nursing helmets, alcohol gel, soap and hand towels, N95 and surgical masks, infrared thermometers, sterile gloves, blood-repellent equipment, goggles, high-density gowns, boots, caps and washable closed shoes. This strategy ensured that the hospital had the recommended work materials (Romero, 21 abr. 2020; La justicia..., 16 jun. 2020) (Interview nurse, Autonomous City of Buenos Aires, June 9, 2021).

The lack of supplies led to a feeling of injustice, due to the lack of protection that this meant: “there should be someone in management who would say: ‘Look, the girls are working without their masks, let’s see, why?’ And justify it: ‘Nursing cannot be without a mask because the patients, until a laboratory comes and until there is a diagnosis, are still considered at risk’” (Interview nurse, La Plata, July 13, 2021).

In the absence of supplies, the nurses organized themselves to buy these protective elements and, in one case, a section coordinator nurse made the purchase and paid out of her pocket. In other places, such as the Hospital de Clínicas in the city of Buenos Aires, an entire floor went on strike to force the authorities into providing nurses with N95 masks, which had only been issued to medical staff or certain sectors. This was reported by a 69-year-old nurse with comorbidities who contracted covid-19 during the performance of her duties:

A nurse is not seen as a doctor. However they all seem to be doing the same things and, in some cases, nurses work harder than doctors. And there is little difference in their knowledge, since a graduated nurse has several years of study. But who stays with the patient? They use the best and the most expensive, earn a lot of money and spend less time with patients. On the other hand, the ones who stay there, body to body, are the nurses. My colleagues have taken money out of their pockets to buy the masks. The light blue ones say they last two to three hours; we use one a day. I work Monday through Friday and I get five. If I wear double I have enough for two and a half days (Interview nurse, Berisso, July 13, 2021).

The existence of obsolete medical equipment was also mentioned. For example, the stand-type sphygmomanometers with air balloon cover and metal foot do not allow keeping distance with the patient, unlike more modern instruments, and some nurses bought them with their own resources (Interview nurse, Berisso, July 13, 2021).

In this context, the death of the nurse Silvio Cufre, on June 8, 2020, caused the National Congress, in its first virtual session during the pandemic, to sanction the so-called Silvio Law 27.548, “Program for the protection of health personnel in the face of the covid-19 coronavirus pandemic”, which establishes biosecurity protocols, equipment and preventive measures, and to regulate it three months after its sanction. The Ministry of Health of the Nation was in charge of establishing a permanent digital advisory team for the protection of health personnel, to establish instruments, guidelines and recommendations (Ministerio de Justicia y Derechos Humanos, 21 mayo 2020).

Despite this regulatory framework, data from the survey conducted a year later regarding the availability of personal protective equipment (PPE) shows that these problems persisted. None of the questions asked about PPE achieved 100% positive responses. Among those surveyed, a large number of cases were found who – between the beginning of the pandemic and June 2021, when the survey was conducted – were still not provided with PPE.

Although this problem was being solved in some places, in others, for example, the use of social masks for the nursing sector persisted:

In my hospital, it still happens that they don’t give us all the medical face masks. The N95 and 3M are given to people who are operating and in surgery. The nurses who enter the covid isolation room have them, but we, the ones in the non-covid isolation rooms, are given the common ones. As we receive talks with infectologists, we know very well that these masks do not protect (Interview nurse, Berisso, July 13, 2021).

During the second wave of infections (from May 2021), there was a lack of other supplies for the direct care of patients in intensive care units (oxygen and sedative medication for intubation). Also, there were unusual accidents or failures: at Cullen hospital in Santa Fe, while oxygen was being loaded, a valve broke and the hose of the main tank was disconnected (Se desconectó..., 4 jun. 2021).

Given the high speed of contagion and the high exposure of the nursing sector, fears were reinforced about increased chances of getting sick due to the lack of supplies. The vaccination campaign against covid-19 was a response that cost many government efforts in a context of global shortages and unequal access. Argentina started it at the end of December 2020, after the arrival of the first vaccine, Sputnik V, developed by the Gamaleya Institute of the Russian Federation (Ministerio de Salud, 29 dic. 2020).

In this case, health teams were given priority for inoculation. Despite some doubts about the vaccine (it was not approved by the WHO), in general, the personnel accepted it because they considered that it prevented undesirable consequences. Likewise, among other measures, the Ministry of Health sought to respond to the voices of the different health sectors, and created the “National Care Plan for Health Workers”, whose resolution (n.987) was published on June 8, 2020 in the *Boletín Oficial*, with the main purpose of “reducing the total number and proportion of health workers infected by covid-19, based on a common strategy in all jurisdictions”. Among other recommendations, he suggested that institutions set up fixed work teams to minimize contacts (Ministerio de Salud, 8 jun. 2020). However, in nursing, one form of moonlighting is the double working day in different facilities, and another is the extension of the schedule by working overtime. Both work as “compensatory” mechanisms for low salaries (Pereyra, Micha, 2016), and the fact that they were not contemplated in the planning made it difficult to implement this recommendation.

Also in spite of this legislation, in response to the question “Do you consider that the opinion and experience of the nursing staff were taken into account in the protocols implemented in your department as a result of the pandemic?” It was shown that the nursing staff considered that “never” or “occasionally” was taken into account in this regard (in the provinces of Buenos Aires, Autonomous City of Buenos Aires and Córdoba, around 70%, and in the provinces of Formosa and Neuquén – also locations where in general there was a high social conflict in the nursing sector –, the answers “never” and “occasionally” reached a peak of over 80%).

Another problems standing out in relation to the implementation of the protocols are the lack of building functionality and the organization of patient and family care.

Regarding the observations that nurses made about the architectural design of the hospitals, it should be noted that, in some cases, the isolation rooms for patients with covid-19 did not have their own bathrooms, which meant that patients wandered between the different rooms. In addition, some storage areas had to be converted into emergency isolation rooms, problems that persisted even when entire rooms were moved to new buildings. For example, in the intensive care room of one of the hospitals there was no place to isolate newborns with covid-19 and therefore, the incubator storage room was used for that purpose (Interview nurse, La Plata, 14 abr. 2021).

Family member’s visits to patients were another problematic issue. If at the beginning of the pandemic total isolation was a painful situation (Pecheny, 2020, p.200), when the guidelines were relaxed, the risks of contagion multiplied. Indeed, given the lack of adequate spaces and the scarce monitoring staff, visiting hours began to compromise hospital inpatients who received relatives that, for example, did not wear their masks properly. The right to visit family members was questioned by some nurses who had to deal with these people and thus put other inpatients at risk. For some of the people interviewed, by contemplating certain rights, they were skipping protocols that exposed them to contagion. It was not, however, a matter of a cold attitude or lack of empathy, but of lowering exposure. As the nurses themselves acknowledged, distancing protocols could not be easily adopted by all patients. This was particularly true in the pediatric, mental health or geriatric sectors. A newborn infected with covid-19 needed the warmth of a hug, regardless of how many times the protocol indicated that the baby should be held, or had needs to be met without conforming to the protocols and their timing. The nurses were willing to break those rules in favor of their patients’ care, even at risk, but there was no way they would accept taking an order from someone who was not in the day-to-day and did not consult with them to implement a rule. In the nursing homes and hospitals of the public mental health network, the situations were complex. These patients had difficulty complying with protective rules such as wearing masks, maintaining social distance, and with holding visits or recreational workshops and rehabilitation therapies. The obstacles to comply with these restrictions had an impact on the contagions. Also, the nurses experienced aggressive situations from the patients who, due to their condition, suffered more the changes that the covid-19 restrictions implied for their lives (Interview with geriatric nurse, Autonomous City of Buenos Aires, June 15, 2021; Interview with nurse, Autonomous City of Buenos Aires, April 23, 2021).

Testimonies, as a whole, they indicate that decisions regarding protocols were made by doctors and management authorities and that in most cases, nurses were not consulted. On the one hand, in general, nurses do not hold decision-making positions or do so only exceptionally. On the other, even being part of crisis committees, their point of view was not taken into account. One nurse reported that “never, so far in the year and a half of the pandemic, we held meetings to organize ourselves with respect to work, to expose our feelings or our ideas” (Interview nurse, Autonomous City of Buenos Aires, June 9, 2021). This revealed a structural issue linked to what some testimonies called “medical hegemony” or “medical model”. With this concept, what the interviewees are trying to explain is that they occupy a subordinate position in the sanitary hierarchies.^5^

## Ways of claiming: from labor unions to the self-convened movement

In Argentina, nurses do not have union representation by occupation. They join different unions according to their field of work (state, private etc.) and according to the legislation that governs it. When they integrate into unions representing broader work collectives, although it can be assumed that they gain greater strength and union negotiation capacity, in fact, they lose the specificity of a highly professionalized sector with very complex particularities in the performance of their working day, especially in pandemics. The clearest example is the case of those representing people working in the state sector, but which includes from cleaning and maintenance workers to administrative staff, and in which nursing is blurred in relation to their specific claims (Aspiazu, 2017; Beliera, 2017). In this regard, it is worth mentioning that even in unions of national scope, the lines of intervention have not been homogeneous. Thus, it is possible to find one that denounces the lack of supplies or demands salary increases at the municipal level while taking another position at the provincial or national level, regardless of the lack of supplies and low salaries.

From that place, during the pandemic, different types of union actions have been detected, some of which were qualified as correct by several people interviewed, especially in the context of the pandemic: “When the scholarship holders were removed, the unions ... had a lot to do with the return of those people.... This is very important. Because we, as colleagues, what can we do? Nothing. You march. But I know that they worked a lot to get them reinstated, because it was not fair [that they were dismissed]” (Interview nurse, La Plata, April 23, 2021).

In parallel, some people explained that the union Association of Public Servants (Asociación de Trabajadores del Estado) intervened to ensure that the young women who joined the program had the corresponding rest. Also, the person interviewed stressed the social function that this organization had fulfilled: “many things were done by the union for people who were isolated. We obtained supplies for them and took them to them. Support was given to both members and non-members” (Interview nurse, La Plata, May 13, 2021). However, these recognitions were rather exceptional. In response to the question “What is your level of satisfaction with the actions of the union/professional association during the pandemic?” the answers are eloquent. The highest level of dissatisfaction is found in the province of Buenos Aires: 75.6% answered that they were dissatisfied or not very satisfied with the union’s performance during the pandemic.

It is not new for the theory of collective action and social movements that there is no single type of motivation for cooperation, but rather that there are chains of cooperation and, in this sense, both altruistic and utilitarian motivations can be distinguished, as well as those imperatives that motivate to do what benefits everyone or in the name of what is considered fair. In this sense, how the union is viewed also affects this motivation. As one nurse summarized:

I don’t particularly like unions. They never end up being independent of the government in power. Union Health leaders have not set foot in a hospital for fifteen years and they talk about a reality they do not know. They negotiate collective bargaining agreements with those who have no idea of what it means to be on duty for twelve or twenty-four or forty-eight hours. They have no idea of the state of the hospitals or of the shortages. Sometimes there is no gauze or tapes to make a cure. Not to mention basic medications such as painkillers. I have a very particular issue with the health unions. I am not affiliated to any of them (Interview nurse, Melchor Romero, June 28, 2021).

Many testimonies underlined the huge distances between the interests of the union delegates and the concrete demands of the nurses: “They never came [to talk with us] they never went [to any hospital]”. You see that they tell you ‘we give you all our support and we are with you.’ Yes, it’s very nice to talk from the outside, but we are the ones who are putting a great effort into it. I am not telling you to come and work alongside us, but come and talk to us, to see what is happening to us, what we are lacking” (Interview nurse, La Plata, May 6, 2021).

At the same time, they underline the non-compliance with their union functions: “It is not that I am against it, but for me it is a waste of money, because they are people who promise things and do not fulfill them” (Interview nurse, Berisso, July 10, 2021).

A profound expression of this unease is the disaffiliation from the union of people who had the interest and conviction in union action and decided to change their plans because they felt completely neglected. A nurse explains it this way: “The union was there and with the pandemic they were all disappeared. We needed the supplies and besides, they had stolen our lockers and accused us of it.” According to the testimony, no union came to her defense: “I was very angry and I left” (Interview nurse, La Plata, May 19, 2021). During the pandemic, many of the people interviewed pointed out that the unions did not reach agreements on salary increases that would allow them to beat the inflation of the period and compensate for the excessive demands of the sector:

It was really the limit. You know that you are being underpaid and you put it up. But in a negotiation, the person who has to defend you accepts a small raise, and this makes you angry. Even those who never complained, start [*sic*] to leave. It was a collective feeling and self-organizations began to take place and, later, [to] make decisions. We joined forces with other hospitals and that allowed us to have more strength to be able to take a stronger measure. Otherwise, it would not have been possible (Interview nurse, Neuquén, June 10, 2021).

Disbelief and unease in this regard were resolved in different ways by the people interviewed. On the one hand, resignation arises: “Nobody is going to defend us. Our boss more than once laughed in our face. She told us: ‘You talk, but nobody is going to defend you.’ And she is right” (Interview nurse, La Plata, Aug. 5, 2021). On the other hand, in individual or small group actions, in the provinces of Buenos Aires, Cordoba and in the city of Buenos Aires, for example, about 40% indicated that they did not get a response to their demands, which, added to the fact that the response obtained was unfavorable, results in approximately 50%. To this must be added the aforementioned claims through judiciary collective protection order.

Finally, there were actions by self-convened movements. One of them was Argentinean self-convened nurses (Enfermeros Autoconvocados de la República Argentina), an organization that emphasizes that keeps its distance from any health union or political party, which consider unrelated to the demands and conditions of the sector. A member of the group said of the first march held in La Plata: “We left the Ministry of Health of La Plata and went to the government house. We were all very happy and proud because we were able to get a lot of support from our sector. The actions were by word of mouth. The problem in nursing is that we were not united” (Interview nurse, La Plata, April 27, 2021).

Another member included symbolic issues in the mobilization: “Although we are demanding a decent salary, we are also asking for social recognition,” and considered that “for the government we are just a number; nursing has not yet woken up, we always think with our hearts, not with our pockets. When we think with our pockets, we look for another job” (Interview nurse, Berisso, July 13, 2021).

In Mar del Plata, at the beginning of 2021, self-convened health care workers demonstrated and highlighted their struggle in defense of their labor rights. They carried out several mobilizations and camped during 21 days on the provincial hospital building. According to local newspapers, with these actions they managed to show the precariousness of their working conditions due to the lack of personnel, the scarce investment in infrastructure and in some basic supplies, the low economic remuneration, the postponement of vacations or rest days, and the transfer to permanent staff and the re-categorization of the personnel, who demand to be recognized as health professionals (Alonso et al., 2022).

In Neuquén, the conflict reached dramatic moments with the roadblocks that the self-convened health personnel carried out to demand better conditions and which were met with violent responses from even the unions themselves. This also happened with self-convened groups in La Plata where, on a more interpersonal level, some interviewees stated: “Nursing carried solo protests [separately from Health unions] and the unions came down on us. In other words, the delegates got angry with me when we organized marches” (Interview nurse, La Plata, April 14, 2021).

In general, both the groups of self-convened health personnel and the trade unions and professional organizations had to face at least two problems in order to channel their demands. On one hand, the lack of media to disseminate their claims, so they used social networks or a few digital media. On the other hand, given the impossibility of demonstrating in public spaces due to the provisions of the ASPO, new protest resources were sought. In the case of the city of Rosario, protests were carried out under the format of “open letter” and cyber protest was also used (Iglesias et al., 2022).

The strike is a less viable option for the nursing sector, since a protest action of this type can be charged with abandonment of person. Therefore, as essential personnel, they must guarantee a minimum service in order to provide care to patients (Aspiazu, 2017; Beliera, 2017). In these cases, the vocation of service and the claim as workers who genuinely require improvements that would not only benefit them, but would also have an impact on society as a whole, including their patients, are put in tension:

I participate in the mobilizations because I think they are necessary. We must go to the streets to demand public and quality health care. I don’t think it is through the unions, but through mobilization in the streets. On many occasions, the unions go back on their word. We don’t want them to take care of our collective bargaining. We want decent working conditions and access for all people. Because most of them are desk bureaucrats; they do not demand or put the same emphasis on any of the issues that also have to do with access to health (Interview nurse, Melchor Romero, April 26, 2021).

In addition to the demands oriented to current needs imposed by the pandemic, two points were key in the agenda promoted by this group: salaries and professional hierarchization.

Low salaries are a historical problem in the sector and it cannot be ignored that this is largely due to the fact that it is a feminized sector. Cultural representations have had a strong influence over time and are difficult to change, which is why discourses that emphasize the “vocation of service” and the “feminine predisposition” continue to be legitimized in relation to nursing. Hence, also, the lack of professional recognition persists (Ramacciotti, Valobra, 2017). Female work is still perceived as exceptional and complementary, and salaries in feminized trades tend to be lower because it is still considered that they do not constitute the contribution sustaining the household. However, this idea is not well harmonized with the data since currently, it is estimated that 85% of the nursing staff is made up of women (Aspiazu, 2017). Our survey also reveals that almost 50% are female breadwinners and that 28% are the main income of the household.

Likewise, 70% of the respondents consider their salary “not at all adequate” or “not very adequate” in relation to the type of tasks they perform. This can be understood by the fact that 25% of the people who responded to the survey are in their main job below $45,000 ARS; 45%, between $45,000 ARS and $60,000 ARS; and 30%, above $60,000 ARS. This becomes even more important when compared to the basic food basket, which, according to Malleville and Noguera (2021, p.173) is 11.6% above the sector salary. Moreover, according to estimates by these authors, “the evolution of comparative remunerations between 2015 and 2020 remained without significant changes for health care workers and nurses” (p.171).

This salary situation was concurrent with our survey results, where we found that 40% confirmed that they have another job in addition to their main job.

In relation to the salary claim, during the pandemic, the national government, by decree, granted monetary compensations. An extraordinary non-remunerative payment of $5,000 ARS was established for the months of April to July 2020 – then extended to the months of August to October of the same year. In April 2021, a new bonus of $6,500 ARS for three months was announced (Malleville, Noguera, 2021, p.174; Argentina, 4 oct. 2020; Argentina, 18 mayo 2021). However, at the time of the interviews, both in the media and in the nurses’ testimonies, it was reported that they had received it in installments or were still waiting for payment. In all cases, they indicated that the amounts were an incentive, but insufficient to compensate their economic situation. Testimonies collected in the province of Catamarca also indicate that the bonus money was used to improve personal protection equipment (Jerez, Reyna, 2022). Finally, the closing of bargaining agreements failed to compensate for the drop in wages due to inflation and unleashed moments of considerable tension against union delegates (Irrumpen..., 8 jun. 2021; Czubaj, 29 jun. 2021).

This panorama becomes even more complex if we take into account that there are contingency scholarship programs attending different emergent situations such as seasonal diseases, vaccination campaigns etc. These scholarships, which are intended for a short period of time, are usually renewed and become a precarious form of employment because they are renewed for a specific period of time and do not imply the recognition of a contractual relationship. This practice is common in public contracting, and in the context of the covid-19 emergency, it turned out to be a strategy to alleviate the lack of structural personnel in some areas, which was enhanced by the contagions and the exit from the system of those suffering from comorbidities due to the disease. In the province of Córdoba, some hiring practices were really rude and inhumane. One testimony relates that those hired for contingency have less than the basics, and that all the extra tasks (weekends, holidays, night work) were not paid (Rodriguez, Pereyra, 2022). Thus, according to the above, nursing has a deficit in the recognition of its professional status, which is crudely expressed in the contracting conditions and in the established salary.

In some political sectors, it is considered that the lack of salary recognition is linked to the lack of training in the sector, hence its consequent professional disqualification, a generalization that seems to take up old representations of nursing, considered a mere empirical knowledge that was learned through practice.

As mentioned above, nursing training is distributed among three types of degrees: bachelor’s degrees (16%), technical degrees (52%) and assistants, who have one-year training and can study without having completed high school. The supply of training for assistants has been significantly reduced, however people with such degrees continue to work (32%) and, as the scale of analysis is reduced, the problem becomes more acute. For example, in the case of the province of La Pampa, 42% of nursing personnel are assistants, a figure that exceeds the national average by ten points and decreases the number of the other two categories, with a greater impact on the technician category, 46%, and also on the bachelor’s degree category, 12%. Thus, the impact of university training and professionalization initiatives was lower depending on the region (Ministerio de Salud, 2020).

However, there is an underreporting of training, due to the fact that, on numerous occasions, nurses are covered by a law on hiring as public employees without being able to enter the hospital career path due to the limitations of the system itself. The case of the city of Buenos Aires is the one that has had the most repercussions. There, law 6035, enacted in 2018, frames nurses as administrative staff, while it recognizes doctors and other related disciplines (psychologists, kinesiologists, nutritionists, among others) as health professionals (Caravaca, Daniel, 2022).

The different legislations that regulate the mode of entry into the labor market in the province of Buenos Aires are no less problematic. As Eliana Aspiazu (2017, p.13) points out,

the multiplicity of norms regulating the occupation and a broad and complex union structure, fragments the representation of nursing workers, limiting the possibility of including specific demands of the occupation in collective bargaining. On the other hand, the deficit in the number of nurses, their overrepresentation in the lower qualified categories, and the relative disadvantage and devaluation of nursing within the health teams, add complexity to the labor panorama of this occupation and place it in a situation of greater precariousness with respect to other workers in the sector.

Our survey also estimates that auxiliary staff tends to decrease, and that technical, undergraduate and postgraduate degrees are on the rise. In fact, in some provinces, the possibility of certifying auxiliary degrees has been suspended since 2016, as in the city and province of Buenos Aires. However, during the health emergency, auxiliary personnel were rehired to cover multiple needs.

## Final considerations

The covid-19 pandemic uncovered long-standing conflicts within the nursing field, such as the lack of funding that has repercussions, among other issues, on the shortage of supplies, long working hours, excessive work demands, lack of recognition that leads to precarious contracts and low salaries. These issues constitute a historical problem of the sector in Argentina to the point that the circle between feminization, low salaries and lack of professional recognition was recognized, demonstrating the way in which class and gender oppressions were knotted in the public labor environment in which nursing is inserted (Ramacciotti, Valobra, 2017).

The increased workload during the health emergency raised the visibility of these problems and made perceptible these long-standing demands. During the pandemic, nurses were constantly exposed to death – other people’s, and potentially their own, on a daily basis. During the period prior to the vaccination campaign, this exposure was much greater, and the only tools for preventing contagion were provided by supplies that were often unavailable, in a context of desperation, fear and anguish in the face of decisions that had to be complied with without their views being heard.

The unions, in general, instead of feeling challenged by the self-convened groups and acting – with a critical sense – adjusting their actions to meet those demands, went out to delegitimize them and to point out that they lacked the altruism expected of their profession, putting their own interests before social ones, without the vocation expected of them.

Besides strong professional commitment and the deep vocation of service that they showed in the face of a health catastrophe such as the one experienced, the nursing staff developed different strategies for making demands. Thus, it should be understood that the requests for supplies, budget and professional recognition have taken on new meanings in this context. Judiciary collective protection orders, community purchases of supplies, complaints through networks and protests marked by limitations and health safeguards show a sector that tries to make itself heard with new repertoires of confrontation in a totally adverse context. If the lack of professional recognition and low salaries are installed as a public agenda of the sector and are receiving some recognition, the pandemic was also a juncture of political opportunities, not only useful to achieve consensus and cultural predisposition to their demands but, fundamentally, to obtain answers that would allow a quantitative-qualitative leap with respect to their critical position. In this sense, although the mobilizing potential of the nursing sector was evidenced, it was also shown that the results of their demands were meager or null.

This health crisis has not been something exceptional and unrepeatable. Different indications lead us to think that dynamic and complex times are ahead and that the role of the sector, given the indispensability of its tasks, will continue to be the mainstay of the health system. The demands in the health sector, and particularly in the nursing sector, should be a guide for the design of public policies in search of a sense of material justice that recognizes the contribution of nursing not only during the pandemic, but undoubtedly for its work during the pandemic itself.

## NOTES

1 In a report on the state of nursing in the world, the World Health Organization highlighted international inequalities in the sector when it comes to tackling covid-19. While Europe and the Americas average around 81 nurses per 10,000 people, Africa has only 8.7 nurses per 10,000 people. With a density of between 20 and 29 nurses per 10,000 inhabitants, Argentina ranks in the lower-middle range in Latin America, along with Paraguay, Peru and Ecuador. In this regard, it is important to take into account the great territorial heterogeneity in terms of the density of nursing technicians and graduates among provinces, which ranges from low densities in Santiago del Estero, Catamarca and Corrientes (5.1, 10.8 and 17.5 per 10,000 inhabitants respectively) to much higher values in Neuquén, Entre Ríos and the city of Buenos Aires (49.9, 50.3 and 61.2 respectively) (East, Laurence, López Mourelo, 2020).

2 The presidents of the most important countries in the hemisphere (Donald Trump, Andrés Manuel López Obrador and Jair Bolsonaro) dismissed the precautions as exaggerated and advised against closures because of their effects on the economy. Boris Johnson and many other world leaders who implemented staggered closures or soft quarantines thought the same (Natanson, mar.-abr. 2021; Nercesian, Cassaglia, Morales Castro, 2021).

3 In Argentina, nursing education is divided into three types of degrees: the bachelor’s degree is a five-year public or private university training program; the technical degrees (*tecnicaturas*), a university undergraduate program (university nurse) or a short course in nursing technical degrees, both of which last three years; and the nursing assistants (*auxiliares*), who have a one-year training and can study without having completed high school.

4 Area comprising the Autonomous City of Buenos Aires, and 24 districts of the province of Buenos Aires.

5 “The concept of hegemonic medical model refers to a set of characteristics: biologicism, a-sociability, a-historicity, a-culturalism, individualism, pragmatic efficiency, curative orientation, asymmetric and subordinated doctor/patient relationship, exclusion of patient knowledge, formalized professionalization, ideological identification with scientific rationality, health/illness as a commodity, tendency to medicalize problems, tendency to split theory and practice” (Menéndez, 2003, p.194).
